# Dr. M’Dowell Once More

**Published:** 1844-11

**Authors:** 


### DR. M'DOWELL ONCE MORE.

When we made the exposure of Dr. M'Dowell's ignorance of diagnosis in our last number, we trusted that we should not again feel called upon to bring him before our readers, nor is it our purpose now to say much more about him; but as he has put forth a pamphlet, which may reach some of our friends, assailing us in language not known among gentlemen, and denying the truth of our report of one of the cases given in our last notice, we trust we shall be excused for devoting a little more space to him, and to his professional and literary pretensions. We challenged him, it will be remembered, to adduce the opinion of a single physician of science, that he had ever cured a case of consumption; and what does he do in reply? He parades certificates from men confessedly and necessarily ignorant of the symptoms of the disease, "*unmedical* men," as he styles them, "that he has cured cases which *he called* consumption;" and this he proclaims "indubitable testimony;" as if the nostrums of every quack in Christendom did not rest upon testimony precisely as indubitable!

The only reply we shall make to his denial of the accuracy of our history of his case, is the following certificate from Dr. Buck, one of the most accomplished young physicians in Louisville. He was present at the autopsy of the lady, to see verified Dr. M'D.'s diagnosis of her case. The following is his testimony:

LOUISVILLE, OCTOBER 28, 1844.

I state, at Dr. Yandell's request, that I understood from Mr. Willard a short time before the death of his wife, what I had repeatedly heard from Dr. Richardson as coming from Mr. W.—that Dr. M'Dowell considered the tuberculous matter as almost, if not entirely, removed from Mrs. W.'s lungs, the cavities having become cicatrized; that the disease from which she then suffered most was *pneumonia;* and that Dr. M'D. believed the mesenteric glands were tuberculous, and very much enlarged. I was present at the *post-mortem* examination, in company with Drs. Drake, Gross, and Richardson. On removing the sternum and ribs, a cavity occupying the entire upper lobe of the right lung, filled with tuberculous matter, was discovered; two or three smaller cavities were found in the left lobe, and the surrounding tissues were studded with tubercles. No cicatrix was discovered in either lobe, nor any sign of pneumonia. Dr. M'D., on seeing the state of the lungs remarked that he had not heard *pectoriloquy* for six months! This I regarded as an expression of his surprise at finding the lungs in such a condition. The mesenteric glands were but little, if at all, enlarged—the promonotory of the spine having been mistaken for their enlargement. (Signed)

J. R. BUCK.

Now the reader will see by this statement, that, far from exaggerating in our report the blunders of Dr. M'Dowell's diagnosis, we suppressed some of the most disgraceful of them. We contented ourselves with showing that he could not detect the presence of tuberculous matter in the lungs, and forebore to exhibit him in the pitiable light of mistaking phthisis for pneumonia, and the promontory of the spine for the mesenteric glands. To such a depth of ignorance, we would hope, there are not many men in an honorable profession capable of descending, and if the exposure of it give him any pain, he has to thank his own folly for it, which induced him to question the veracity of our statement. We will only consume the time of the reader to remark further, that this pamphlet of Dr. M'D.'s shows him to be as incapable of writing the English language, or even of spelling common English words, as Dr. Buck has proved him to be of recognising a case of consumption when he has one before him. But, if he has not learned to write, or to spell, or to distinguish consumption from pneumonia, or the mesenteric glands from the back-bone, he may console himself with having studied figures to some purpose, and with understanding how to run up long bills against patients after other physicians have given them up to die. His art in this respect is consummate, as numerous cases, well known in this city, fully attest. Of such an author and practitioner, whose appeal has now been made from the profession to the people, we cannot be expected to have anything more to say.

